# Visual Adaptations for Mate Detection in the Male Carpenter Bee *Xylocopa tenuiscapa*

**DOI:** 10.1371/journal.pone.0168452

**Published:** 2017-01-20

**Authors:** Hema Somanathan, Renee Maria Borges, Eric J. Warrant, Almut Kelber

**Affiliations:** 1 IISER TVM Centre for Research and Education in Ecology and Evolution (ICREEE), School of Biology, Indian Institute of Science Education and Research Thiruvananthapuram, Kerala, India; 2 Centre for Ecological Sciences, Indian Institute of Science, Bangalore, India; 3 Lund Vision Group, Department for Biology, Lund University, Lund, Sweden; University of California San Diego, UNITED STATES

## Abstract

Sexual dimorphism in eye structure is attributed to sexual selection in animals that employ vision for locating mates. In many male insects, large eyes and eye regions of higher acuity are believed to facilitate the location of females. Here, we compare various features of male and female eyes in three sympatric carpenter bee species, which include two diurnal species (*Xylocopa tenuiscapa* and *X*. *leucothorax*) as well as a nocturnal species (*X*. *tranquebarica*). In *X*. *tenuiscapa*, males have larger eyes than females, while in the nocturnal *X*. *tranquebarica*, males have slightly smaller eyes and in *X*. *leucothorax*, the eyes are of similar size in both sexes. *X*. *tenuiscapa* males detect females by perching near nest sites (resource defence) or along fly-ways and other open areas with good visibility. Males of the other two species search for females by patrolling. We postulate that the larger eyes of male *X*. *tenuiscapa* are beneficial to their mode of mate detection since perching males may benefit from a larger visual area of high resolution detecting moving stimuli across the sky, and which may be germane to the more social and gregarious nesting behaviour of this species, compared to the other solitary bees. We tested the performance of the eyes of male *X*. *tenuiscapa* behaviourally and find that a perching male can detect a flying female at a distance of 20 m, which darkens the visual field of a single ommatidium by just 2%. This, together with the bee’s high spatial resolution permits detection of moving stimuli at least as well or even better than achieved by honey bee drones.

## Introduction

Sexual dimorphism in animals is commonly thought to evolve through male–male competition or female choice [1–6]. Studies that link sexual dimorphism in insects to mating behaviours usually examine morphological traits such as size, colour, ornamentation and pheromone glands, attributing exaggerated traits to the extreme competition for mates [7–11]. However, functional traits in sensory organs, which improve the detection of mates, are also under selection (e.g. [12, 13]). Several studies have reported associations between mating behaviours and functional characteristics in insects. In bees, these include (a) the size of mesosomal glands (source of sex pheromones) which can be larger in males that engage in non-territorial patrolling, since in this case sex pheromones have to be broadcast over larger areas [14], (b) dimorphism in the antennae and the antennal lobes, where macroglomeruli are the male-specific projection sites of pheromone receptors [15], (c) colour dimorphism that provides a visual cue for mate recognition (e.g. [16]), and (d) eye size that may be larger in males to aid in the visual location of females (e.g. [17,18]).

Generally speaking, eyes are sexually dimorphic in insects that search visually for mates, with adaptations in male eyes to improve detection of females. Such adaptations have been studied in many groups including butterflies (e.g. [19]), mayflies [20] houseflies [21], hover flies [22], and stalk-eyed flies [23]. This sexual dimorphism sometimes involves differences in spectral sensitivity (for instance in butterflies (e.g. [19, 24]), flies [25] and honeybees [26]), but more commonly it involves differences in spatial resolution and sensitivity. In these latter cases, the compound eyes of males differ from those of females by having ‘acute zones’ or ‘bright zones’ that permit effective detection and tracking of females (e.g. [27]). Acute zones are flattened eye regions whose ommatidia have enlarged facet lenses, an adaptation for high sensitivity, as well as small inter-ommatidial angles, an adaptation for high spatial resolution [21, 28]. Sometimes acute zones become so dominating that they lead to the division of each eye into two sub-eyes, one of which becomes entirely devoted to sexual behaviours, as has occurred in some species of mayflies and march flies [29]. Bright zones are similar to acute zones, but sacrifice spatial resolution in favour of even higher sensitivity by allowing larger inter-ommatidial angles, thus maximising contrast sensitivity for small targets, especially in dimmer light [30, 31]. Male-specific acute zones have been studied extensively in flies [21, 29, 32] and in drones of the European honey bee *Apis mellifera* [17, 26, 33], but have more recently also been described for Asian honey bees [18] and several species of bumble bees [15]. In *A*. *mellifera*, drones congregate at sites visited by virgin females during nuptial flights [34, 35]. *A*. *mellifera* drones have an acute zone in the upper third of the eye, with considerably larger facets and smaller inter-ommatidial angles compared to the remainder of the eye [26]. The contrast sensitivity of the receptors in this eye region enables a drone to detect a queen against the sky, even when she only covers a small part of the visual field of a single ommatidium, reducing the photon catch in that particular ommatidium by only 6 to 8% [36].

The largely tropical carpenter bees of the genus *Xylocopa* (Family: Apidae), are an interesting group to examine the adaptive value of visual capabilities and modifications in the context of mating behaviours for two reasons: this large genus consists of diurnal and nocturnal species that have specific visual adaptations matched to their varied lifestyles [37, 38], and they display diverse mating strategies [39]. In a phylogenetic analysis of the evolution of mating behaviours in 38 species of carpenter bees, Leys and Hogendoorn [39] determined that resource defence behaviour, small mesosomal gland size and monomorphic sexes constituted the ancestral state. Three male mate location strategies have been described in *Xylocopa* [40]: (a) female defence, in which males perform patrolling flights at the entrance of nests, (b) resource defence, in which males patrol feeding sites that females visit, and (c) male dominance polygyny, in which males patrol territories that do not include nests or floral resources. In all reports of mate location in carpenter bees, males have been described as flying and patrolling along a route, or as cruising in a defined territory containing resources or landmarks [40, 41]. These flights are likely to be energy-consuming, and in one case, males of *X*. *nigrocincta* have been observed to perform nectar-concentrating behaviour by regurgitating a nectar droplet to allow the water in the nectar to evaporate thereby reducing load to improve flight efficiency prior to such patrolling flights [42]. Upon spotting a female, a patrolling male will rapidly approach and face her, starting a courtship flight while spiralling skywards, as described for instance in *X*. *torrida* [43].

In this study, we report a new mate location strategy in the Indian carpenter bee *X*. *tenuiscapa* and compare it to the mating strategies of two sympatric congeners, the diurnal *X*. *leucothorax* and the truly nocturnal *X*. *tranquebarica*. Next, for *X*. *tenuiscapa*, we determine the ability of males to detect females from a distance. Finally, we optically determine the spatial resolution of the eye of *X*. *tenuiscapa* and link mate search behaviour to eye morphology in males of these three species.

## Methods and Materials

### Study site, species and field observations of mating strategies in *Xylocopa*

Carpenter bees were studied in Bhimashankar Wildlife Sanctuary (19°21’-19°11’N, 73°31’-73°37’E; elevation 900 m), Maharashtra State, in the Western Ghats of India. Permissions for field studies were granted to RMB by the Maharashtra State Forest Department and the study did not involve endangered or protected species. The habitat consists of stunted crest vegetation, tall semi-evergreen and moist deciduous valley forests, open grassy patches, and agricultural holdings. The biology and flight activity of these bees were detailed earlier [44, 45]. Carpenter bees build nest tunnels with a single entrance within dead tree trunks or limbs. They are typically solitary bees, a single tree log or limb containing a single nesting female or an aggregation of nesting females. Nest aggregations of *X*. *tenuiscapa* are typically larger (up to 30 nests in our study site) than those of the other two species (1 to 5 nests). Observations of mate location behaviours were made at nest sites as well as other locations for all three species. The activity of males was recorded during the dry season between February and March 2007 and 2008, which was the annual peak in mating and foraging activities in these bees that also overlaps with peaks in community flowering.

### Behavioural tests of female detection distances by *X*. *tenuiscapa males*

To estimate the distance over which a perching male can detect and follow a flying female, we observed males at perches away from their nest sites. Male *X*. *tenuiscapa* choose to alight and perch on vantage points, such as bare tree branches or single-storey roof top structures, from where they attempt to detect and pursue passing females in flight. Observations of matings were rare, since very few approaches by males culminate in successful mating. Perched males were observed to track and follow moving objects across the sky only to return to the perch when an object was not a female. Experiments to measure detection distances by perched males involved throwing bee-sized stones in an arc close to perching males to simulate a flying female bee. A total of 220 trials were performed at 5 perch locations on trees for at least 3 and up to 5 focal perched males. We tested the response of perching males to stones of known sizes that we threw by hand. Stone diameter was measured with Vernier calipers to the nearest 0.1 mm (ranging from 9 to 32 mm at their largest extent), and when thrown, passed by in front or behind a perched male bee. From the trajectory of each stone, observers (four observers were involved simultaneously) estimated the closest distance from the stone to the male bee (between 1 and 6 m) using a horizontal grid as a reference. The error in this visual determination of distance was estimated to be less than ±0.2 m. A response in a trial received a binary score of 1 if the male took flight or 0 if it did not move from the perch in response to the stone. Using the estimation of the closest distance *d* (mm) of the stone to the perching male bee together with the stone’s diameter *s* (mm), we calculated the stone’s angular extent *θ* at the eye of the bee (*θ* = 180*s*/*πd*, in degrees). We next determined the relationship between the stone’s angular extent and the probability that it elicited a response from the male. The error in our distance estimations (±0.2 m) will have the greatest impact on the precision of our calculation of *θ* for the smallest values of *θ* giving a chasing response (≤0.3°) at the closest distances of the stone to the male bee (where ±0.2 m is a greater proportion of this distance). The shortest distance for which a stone that subtended 0.3° or less initiated a chasing response was 2.7 m. This stone subtended 0.30°. At 2.7+0.2 m, the stone subtended 0.28°, and at 2.7–0.2 m it subtended 0.32°, that is, the error in *θ* is ±0.02° (i.e. ±7%). For greater distances and/or larger values of *θ*, the error will always be smaller than ±7%, indicating that our method to visually estimate distance—despite its inherent level of uncertainty—does not significantly impact on the precision of our calculations of the stone’s angular extent *θ*.

### Eye morphology and optics

Briefly described here are the standard procedures that were followed to map interommatidial angles in the frontal part of the visual field [21, 46, 47]. An immobilised bee was mounted at the centre of curvature of a Leitz goniometer with the flat posterior eye edge parallel to the plane of the goniometer stage, and placed beneath an optical apparatus consisting of a Canon MD150 digital video camcorder and an inverted Hasselblad Distagon 1:3.5 60 mm camera objective (with 80 mm back focal distance). This optical apparatus acted as a microscope that allowed single images to be captured from the Canon camcorder.

The bee’s head was positioned such that the three goniometer axes were lined up with the dorsal–ventral (yaw), anterior–posterior (roll), and left–right (pitch) axes, respectively, of the bee’s head (i.e. the bee’s head was at the centre of curvature of the goniometer). With the stage horizontal, both eyes then looked vertically upwards into the rear lens of the Hasselblad objective. The eyes were illuminated with a small hand-held LED torch, allowing us to see the ommatidia looking upwards into the microscope as a dark pseudopupil. We tilted the bee’s head on the stage of the goniometer in defined angular steps of latitude and longitude, with latitude = 0° and longitude = 0° (0°, 0°) defined as the anterior orientation, and took a series of images of the dark pseudopupil in the left eye at 10° intervals of latitude and longitude. Barium sulphate powder was sprinkled lightly on the eye to provide landmarks. Due to the structure of the apparatus we could not go beyond latitudes of +70° or -70° or a longitude of 100°. Hence, our observations of the appearance and location of the pseudopupil were restricted to this part of the eye, in which the highest resolution and sensitivity is found for most bees.

From each image, we determined the coordinates and diameter of the facet at the centre of the pseudopupil. Using established formulae that correct for latitude distortions in the projection [21], we calculated the average local interommatidial angle Δφ for each combination of latitude and longitude. These data were plotted on a sphere representing three-dimensional space around the animal, and contours were interpolated to connect regions of space viewed by parts of the eye with the same Δφ. We also prepared plots of facet diameter *D* and the eye parameter *p* at each point in the eye. The eye parameter, the product of *D* and Δφ (*p* = *D*Δφ μm rad), is an indicator of the evolutionary trade-off between resolution and sensitivity of an eye, both between species, between sexes and between eye regions. Small values of *p* generally mean that the eye, or that region of the eye, maximizes acuity at the expense of sensitivity [48].

For measurements and illustrations of the eyes, we obtained scanning electron micrographs (Hitachi SU3500) of the heads of two preserved male and female specimens of each species, using standard procedures.

## Results

### Mate location strategies in male carpenter bees

**Fig 1:**
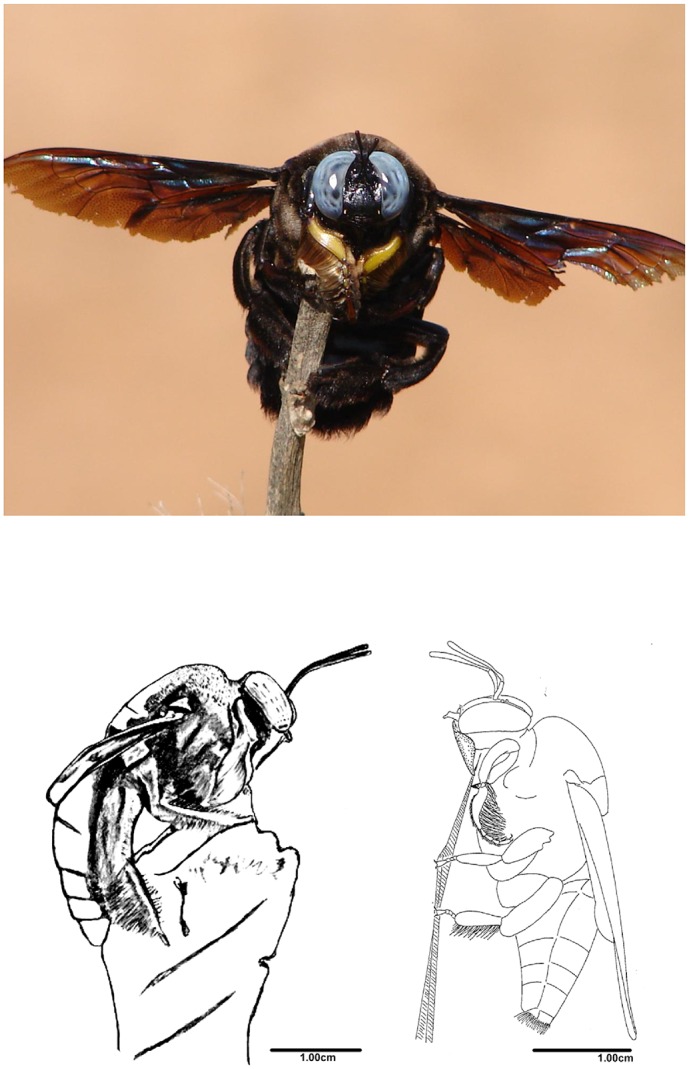
Upper image: A perching male *X*. *tenuiscapa*. (Photo courtesy: U.V. Rane). Lower images, left: sketch of a perching male *X*. *tenuiscapa*; note the spread wings. Right: resting *X*. *tenuiscapa* in which the wings are not spread (modified from [50]).

**Fig 2:**
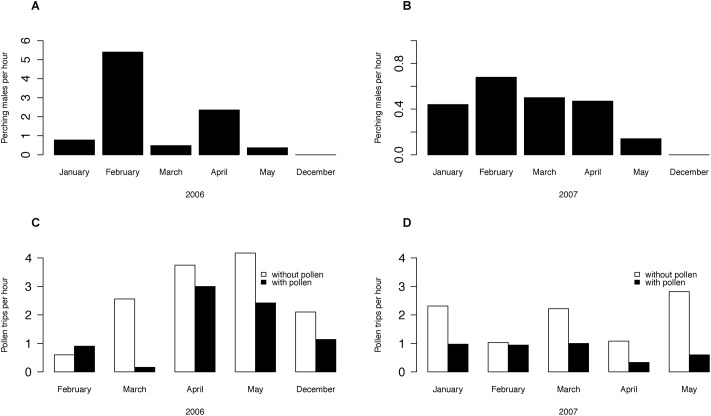
Seasonal changes in males perching outside nests and female foragers returning to nests. Number of males perching outside nest sites (n = 7) peaked in February 2006 and March 2007 (A, B) when the number of unmated females was high. The number of females returning to the nest with pollen (C, D) was highest in May in 2006 (n = 251 trips from 7 nests) and 2007 (n = 206 trips from 6 nests).

*X*. *tenuiscapa*: Males were observed to perch on branches of shrubs and trees for extended durations (up to 25 minutes), in a typical posture with extended wings and upright antennae (Fig 1). Males perched in close proximity to nest sites early in the dry season (February; Fig 2). Perches were located either in front of or to one side of the nest log, about 1–3 m meters away, which increased a male’s chances of encountering unmated females flying into or out of the nest. However, later during the dry season (April and May), males preferred to perch at non-nest site locations, especially in exposed positions atop single-storey buildings or on bare branches of trees, a behaviour that falls under the general description of hill-topping [49]. This shift in mate-search location during the late dry season coincides with the period when most females have been mated, as evidenced by a large number of females returning to their nests with pollen loads, which they use to provision individual brood cells, into each of which an egg is laid (Fig 2).

*X*. *leucothorax*: In this sympatric diurnal species, males neither perched outside nests nor hill-topped at non-nest locations. Instead, male *X*. *leucothorax* engaged in the aerial patrolling of a small area, roughly measuring 2 x 2 m in the sub-canopy region, usually between trees or in a light gap. Aerial defence involved flying back and forth over a small clearing, or a gap in the sub-canopy, with intermittent hovering and turning 360° around the horizontal body axis.

*X*. *tranquebarica*: Males of this nocturnal species are identifiable from the prominent yellow clypeus and were seen flying around and patrolling flowering bushes that females visit shortly after sunset. But unlike *X*. *tenuiscapa*, and despite many hours of observation across two years, males were never observed to perch near nests. An opened-up nest was found to contain both males (*n* = 8) and females (*n* = 14), and males continue to use female nests long after eclosing.

### Behavioural tests to estimate detection distances in *X*. *tenuiscapa* males

Males perching in hill-top positions very often chased flying conspecifics, other insect species, birds and even stones thrown at a distance. After pursuing the aerial object for a short distance, the male would presumably detect its error if the object was not a female and return to the perch. In only three cases did we observe successful mating. On one occasion the male reached the female and the pair started a spiralling upward flight until they were no longer discernible to the human eye. This kind of courtship behaviour in free flight has been earlier described for carpenter bees including *X*. *tenuiscapa*, as well as in other bees [40, 50]. In one case, the male reacted to a female flying by at a distance of 20 m (a distance we could measure accurately from surrounding landmarks). Assuming a body length of 30 mm for the female, we can calculate the angle this female subtended at the male’s eye when he saw her and initiated pursuit as 0.086°or 5'.

**Fig 3:**
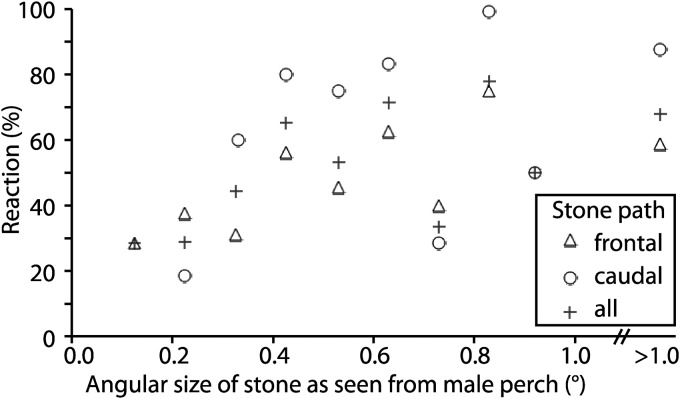
Response of *X*. *tenuiscapa* males to stones of different sizes. Stones of known size were thrown by hand; the smallest distance to the perching male was determined to the nearest ±20 cm and the stone’s angular extent was calculated. Frontal: stone passed in front of the male; caudal: stone passed caudally to the male; all: average of all males, irrespective of stone path.

Stones thrown at perching males passed in front 133 times and 90 times behind them (n = 223 trials). Males initiated flight and followed stones in about 48% of the trials (in 54% of the trials thrown behind, and 46% of trial in front). Stones had angular extents between 0.15° and 3.6° when seen from the bee’s perching position, but on most occasions, the angular size of the stone was between 0.2° and 1°. We therefore defined size bins from 0.1° to 0.2°, 0.2° to 0.3°, and so on (Fig 3). Clearly, males reacted occasionally even in response to very small stimuli, the smallest being a response to a stone with an angular size of 0.19°. Even with large stones, focal males did not respond in more than 80% of all trials (Fig 3 and S1 Table). At least two reasons may account for this: first, the males may not always be alert and responsive, and second, some stones may have traversed at speeds greater than the speed of a female flying at some distance. Initially, we tried to catapult metal balls using a slingshot, but males never reacted to these, probably because they moved faster than stones thrown by hand. Therefore, we are likely underestimating the male’s ability to react to small stimuli, even more so due to the fact that we always measured the largest radius of irregular stones; thus the areas seen by the males were always smaller than πr^2^.

### Sexual dimorphism in eye morphology

**Fig 4:**
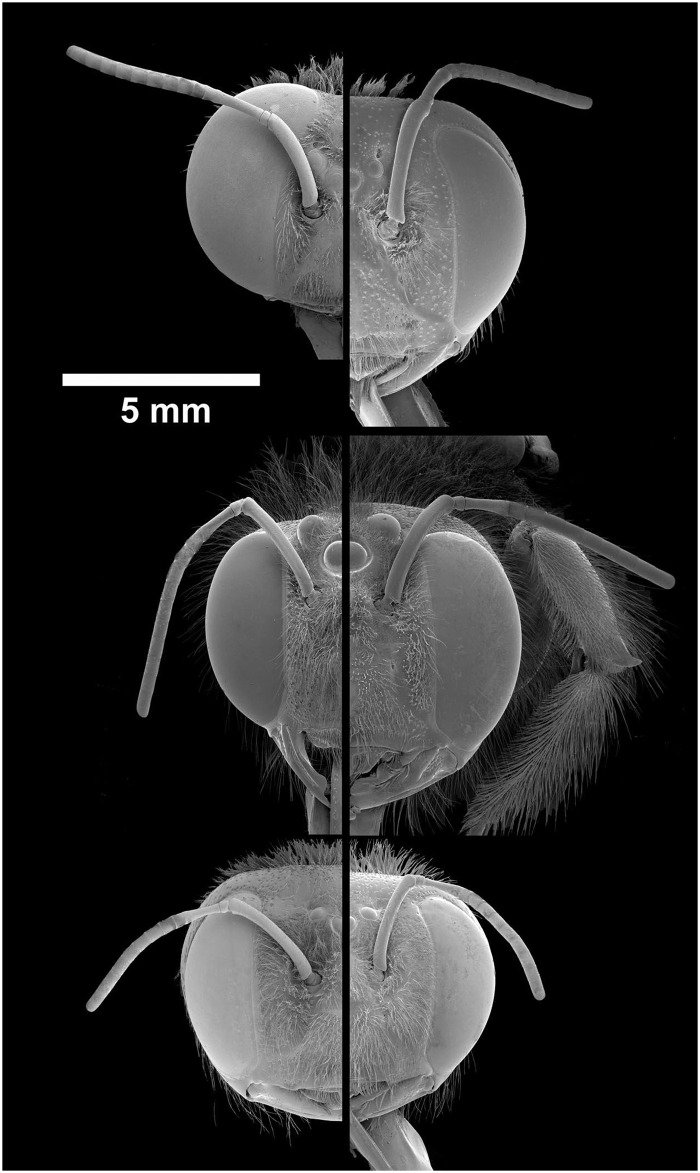
Half heads of *X*. *tenuiscapa*, *X*. *tranquebarica and X*. *leucothorax*, from top to bottom, with left column: males; right column: females.

In all three species, intertegular width (between the wing bases) was larger in males than in females (Table 1). However, relative eye size differed considerably, being similar between the sexes only in *X*. *leucothorax*. In *X*. *tranquebarica*, males have slightly smaller eyes than females (Table 1, Fig 4). In both the above species, head shape is similar between the sexes, but males have fewer ommatidia than females.

**Table 1:** Comparison of optical parameters in male and female carpenter bees. Optical parameters were estimated for two males and 3–4 females (data from [37, 38]) of the three species *Xylocopa* (mean ± sd). Intertegular width refers to the distance between the two wing bases and is a proxy for body size. The number of facets was determined in one eye of a male and a female of each species. Five measurements of facet diameters per bee were made.

	*X*. *tenuiscapa*		*X*. *leucothorax*		*X*. *tranquebarica*	
	Female	Male	Female	Male	Female	Male
Intertegular width (mm)	8.8 ± 0.4	12.7 ± 0.1	7.1 ± 0.7	10.0 ± 0.1	7.5 ± 0.8	9.6 ± 1.1
Eye length (mm)	5.7 ± 0.3	6.1 ± 0.1	6.7 ± 0.3	5.1 ± 0.1	4.5 ± 0.6	4.8 ± 0.4
Median ocellus diameter (mm)	0.5 ± 0.0	0.6 ± 0.3	1.0 ± 0.1	0.9 ± 0.0	0.4 ± 0.0	0.4 ± 0.0
Facet number	15994	15751	18804	15511	12716	11331
Maximal facet diameter (μm)	37.3± 3.9	48±1.1	38.7± 1.3	40±1.2	34.2± 2.5	35±0.1

**Fig 5:**
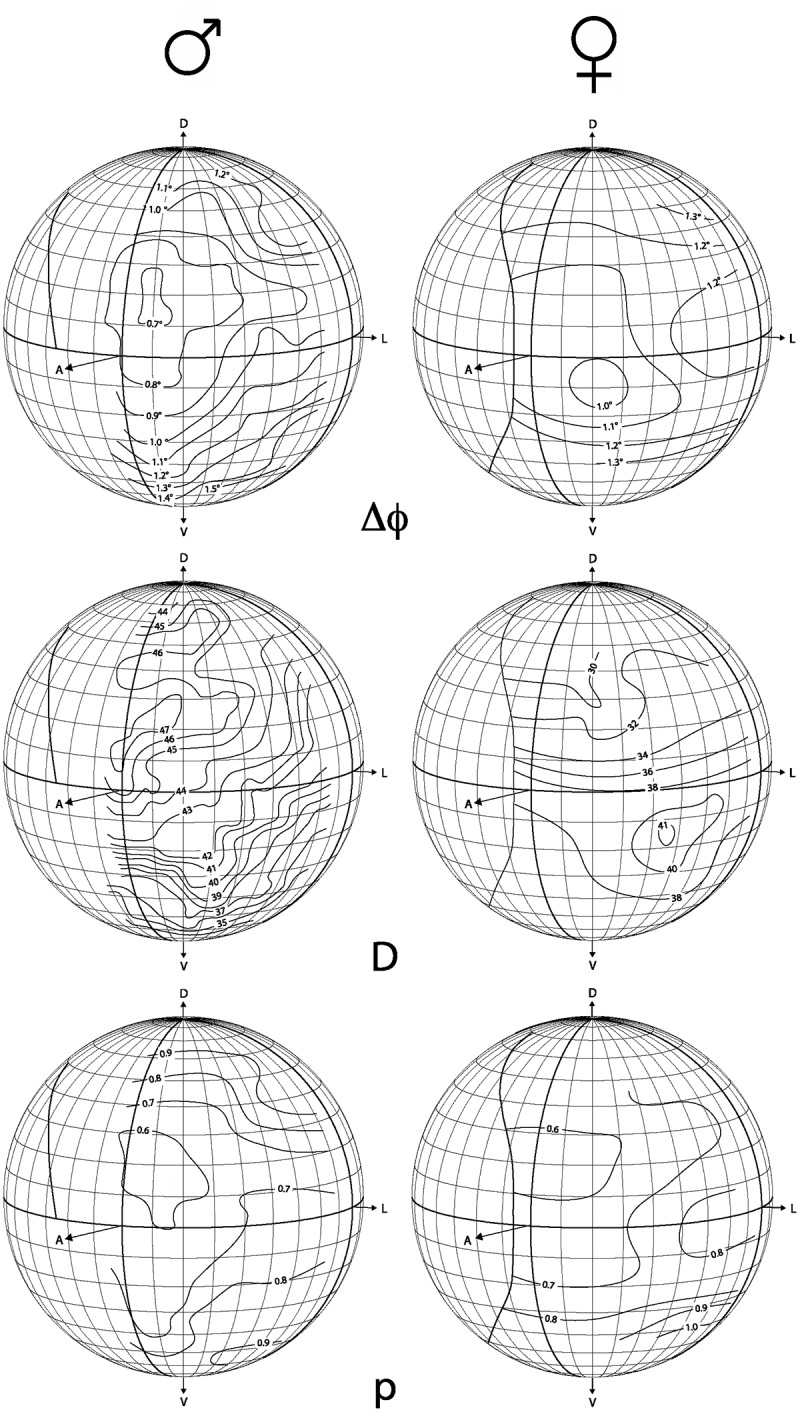
Eye maps of male and female *X*. *tenuiscapa*, showing interommatidial angles (ΔΦ), facet diameters (*D*) and eye parameters (*p*). Female maps are taken from our earlier study [38].

Compared to the other two species, sexual dimorphism of the head is very obvious in *X*. *tenuiscapa*. While males have larger eyes than females, both sexes have similar number of facets which explains the larger facets in males. On average, the largest facets have a diameter of 48 μm in male eyes, but only 37 μm in female eyes (Table 1). In females, the largest facets are looking forwards and slightly downwards, with interommatidial angles of 1° (Fig 5). In males, the largest facets are combined with small interommatidial angles down to 0.7°, and are found within an acute zone located in the eye’s frontal-dorsal visual field (with highest resolution approximately 20° above the eye horizon). These angles are measured with the back of the eyes oriented vertically. Given that perching males have the head tipped backwards by about 30° (see Fig 1), their acute zones actually view the visual world at a physical elevation of about 50°.

## Discussion

### Mate location behaviour in *X*. *tenuiscapa*

To the best of our knowledge, the perching we observed in *X*. *tenuiscapa* has not previously been described as a mate location strategy in any *Xylocopa* species. The mate searching behaviour of 15 other species of *Xylocopa* is described in the literature (see S2 Table), but none of these has been described as perching. Perching is, however, known from bees in other genera, e.g. bumblebees [51] and orchid bees [52]. When Osten [50] observed males of *X*. *tenuiscapa* on a deserted runway in Sri Lanka, he saw them patrolling 3 m above ground between bushes of an important nectar source (*Calotropis gigantea* L.). Upon spotting a female, the male would rapidly approach her, face her, and start a courtship flight, similar to the flight described for *X*. *torrida* [43]. In addition to describing mating behaviour, Osten also documented the resting posture of male *X*. *tenuiscapa* ([50]; Fig 1C). He mentions that males, after a period of patrolling, would sit down on a twig and rest. Did he mistake perching males as resting? In his drawing of a resting male (Fig 1C), the posture is different from the posture of perching males that we observed (Fig 1A and 1B), which makes a misunderstanding unlikely. The resting male drawn by Osten sits with the wings closely attached to the abdomen, while perching males sit with both wings at right angles to the body’s long axis. We suggest that our observations of perching males together with Osten's observation of patrolling males strongly suggest plasticity in mate search behaviour in *X*. *tenuiscapa*.

Moreover, *X*. *tenuiscapa* males seem to vary in their mate searching behaviour seasonally by engaging in nest site defence early in the season, when females that nest in large aggregations are unmated largely, while later in the season, males largely employ non-resource defence that likely enhances the chances of encountering unmated females from multiple nests. These behavioural differences in males indicate that mate searching behaviour is influenced both by the spatial distribution and reproductive status of females. Differences in mate location strategies of males have earlier been reported between species as well as between conspecifics [53–55]. During periods when many virgin females are expected to emerge from nests, we observed perching males at nest sites while later in the season, we mostly saw males hill-topping at non-resource locations. In a range of social and solitary bee species, females generally mate soon after emergence from the natal cell and rapidly lose receptivity, following which provisioning and egg laying usually occurs [56]. Hence, males likely enhance their reproductive success by maximising access to receptive females. Male fitness may be maximised by defending sites closer to nest entrances early in the season, while later in the season, non-resource sites are likely to be more profitable. Plasticity in mating behaviour at all these different levels can have fitness benefits via a reduction in competition for display sites and mates [57–59]. However, from our study, it is not possible to conclude whether the same males switch their mate search tactic between early and late seasons or whether early and late emerging males employ different strategies: nest defence or non-resource defence. Mate searching has been well studied across animal groups and the optimal male mate searching strategy depends on multiple factors such as habitat type, density and dispersion of females, season and male–female sex ratios [60, 61]. Considerable interspecific diversity in the selection of rendezvous sites and male mating behaviour has been reported in bees and butterflies [62, 63].

### Inter-specific differences in mate location behaviours in male carpenter bees

Our study suggests that males of the three sympatric *Xylocopa* species differ in mate searching behaviours. *X*. *tenuiscapa* and *X*. *leucothorax* overlap in their diel activity periods; hence the differences in their mate searching behaviours may be attributed to avoiding interspecific interactions by reducing spatial overlap at mate-search locations. Occasionally, we have observed antagonistic interactions between these two species, with *X*. *tenuiscapa* males chasing *X*. *leucothorax* at flowering plants. *X*. *tenuiscapa*, is larger than *X*. *leucothorax* and much more aggressive in chasing both conspecific and interspecific intruders. The aggressiveness displayed by *X*. *tenuiscapa* males may also be attributed to the fact that chasing other ‘moving objects’ might increase their chances of detecting relevant stimuli, i.e. a conspecific female moving across the sky, which is more likely in the absence of other distracting stimuli. An alternate explanation for the interspecific differences between the bees is connected with the spatial resolution of the male eyes and the fact that they might only be able to discriminate between moving objects across the sky on closer inspection and possibly more reliably when visual cues are coupled with female sex pheromones. A third explanation might involve differences in thermoregulatory capabilities between the males of *X*. *tenuiscapa* and *X*. *leucothorax* that may determine whether they employ perching or patrolling as mate detection tactics. On the other hand, while males of the truly nocturnal *X*. *tranquebarica* have been observed leaving nests at night, they were never seen patrolling the vicinity of nest sites or patrolling and perching at non-resource locations during many hours of observations during moonlit or moonless nights in our study site. Low light intensity may render these types of mating strategies ineffective, and the large mesosomal glands of the species suggest a primarily olfactory mate location strategy [39]. However, *X*. *tranquebarica* males have been observed patrolling flowering shrubs that females visit shortly after sunset, though it is not known if this is restricted to the brighter periods of the night.

As in bees, perching and patrolling have been identified as two alternate mate detection tactics in 16 butterfly species [63]. In butterflies, it was hypothesised that in species where the males perch, a larger visual field as well as high acuity throughout the eye or in localised regions of the eye, will be beneficial for maximising detection of moving stimuli compared to species that patrol. However, although males of all 16 species had larger eyes relative to females (after controlling for differences in body size between the sexes), eye size alone did not predict patrolling versus perching as mate location tactics employed by males.

### Linking eye morphology and visual capabilities to mate search behaviours

In honeybee drones, the dorsal half of the eye has ommatidia with large facets (with up to 41 μm diameter) and small interommatidial angles (down to 1°), which form an acute zone of increased light capture and enhanced contrast sensitivity [26]. The smallest acceptance angles measured electrophysiologically in drones are as small as 1.28° [64, 65, 66]. In behavioural experiments, Vallet and Coles [36] attracted drone honeybees to dummy females and observed that drones were able to detect objects subtending 0.41° in the visual field, thereby reducing light flux in a single ommatidium by as little as 8%.

In *X*. *tenuiscapa*, a larger species with much larger eyes, we have observed a male reacting to a passing female covering less than 0.1° of his visual field. In our experiments, the smallest angle of a stone to which a male responded was 0.19°. We found the smallest interommatidial angles in the acute zone of males to be 0.7° (Fig 5). Assuming a matching acceptance angle of 0.7°, we estimate that a female flying at a distance of 20 m from a perching male would darken the visual field of a single ommatidium by only 2%. In our experiments, the smallest stones eliciting a response from males are estimated to darken the visual field of a single ommatidium by about 7%. Thus, our results show that carpenter bees have similar, if not higher, contrast sensitivity than honeybee drones. In combination with their higher spatial resolution, this would allow *X*. *tenuiscapa* to detect passing females at a considerably greater distance than honey bee drones [36].

Although mate search in *X*. *tenuiscapa* appears to be primarily a visually-guided behaviour, it is likely that sex pheromones play a role at a closer range. Males that approach a flying female, will often give up the pursuit when they are very close to the female, possibly because of a weak or altered female sex-pheromone signature which the males perceive. In fact, Leys and Hogendoorn [39] who compared male eye size and mesosomal gland size, found trade-offs between investment in eye and mesosomal gland size. Out of the 44 carpenter bee species for which male eye sizes and gland sizes were available [39], enlarged eyes were associated with small mesosomal glands in 5 species, while large glands was associated with normal-sized eyes (with no sexual dimorphism in eye size) in 29 species including the nocturnal *X*. *tranquebarica*.

## Conclusions

We have observed *X*. *tenuiscapa* males perching at nest sites and at hill-top sites unrelated to resources. Osten [50] observed males of the same species patrolling small territories close to important foraging sites. It is unclear whether perching or patrolling—and thus detecting a mate while flying or sitting—puts higher demands on an insect's visual system, and it is also unclear whether the choice of the perching site or patrolling territory or route (e.g. nest sites, foraging sites or sites unrelated to resources) favours different visual adaptations. However, since possessing large eyes is costly, it is clear that only species that primarily use vision to detect mates can invest in large eyes [67]. We assume that males of other *Xylocopa* species with sexually dimorphic eyes are also likely to use vision for mate detection. In other species of *Xylocopa* without sexually dimorphic eyes, such as the other two species investigated in our study (*X*. *tranquebarica* and *X*. *leucothorax)*, mate-searching strategies are instead likely to be primarily based on olfaction. Hence, we argue that a more comprehensive classification of mate-searching strategies, should not solely be made depending on whether a species defends resource or non-resource locations, or by patrolling sites, but should also take the primary sensory modality used for mate detection into account.

## Supporting Information

S1 Table Field experiments estimating the reaction of perching male *X*. *tenuiscapa* to stones thrown of known sizes. (XLSX)

S2 Table Mate location behaviour and associated morphological adaptations reported in male carpenter bees. (DOCX)
