# Supplementary material for: Visual Adaptations for Mate Detection in the Male Carpenter Bee Xylocopa tenuiscapa
Source: PLoS One. 2017 Jan 20;12(1):e0168452. doi: 10.1371/journal.pone.0168452 (PMC5249068; doi:10.1371/journal.pone.0168452)
Supplement: S2 Table — (DOCX) [file pone.0168452.s002.docx]

**S2 Table.**  Mate location behaviour and associated morphological adaptations reported in male carpenter bees.

| **Species** | **Male behaviour** | **Male morphological adaptations** | **Reference** |
| --- | --- | --- | --- |
| *X. aeratus* | Hovering males engage in non-territorial patrolling near nests or flowers. Males defend nests containing unmated females by flying loops of 1 m. This can be directed at several nests, and other males are chased. Males also perform non-resource territoriality (hill-topping) at tree tops, above bushes or rocks while flying in figure- of-eight loops or while flying along linear paths in small areas about 2–5 m in diameter. | Rudimentary mesosomal glands | [1] |
| *X. bombylans* | Non-resource territoriality | Well-developed mesosomal glands | [1] |
| *X. californica arizonensis* | Males hover close to entrances of nests and chase away other males. Males patrol and approach females at non-territorial locations such as food plants. |  | [2 ] |
| *X. fimbriata* | Males hover and turn around own body axis at non-resource sites; scent marking is also reported. | Secretions from the mesosomal glands | [3 , 4 ] |
| *X. flavorufa* | Males circle tops of bushes in horizontal figure of eight flight paths with abdomen pointing inwards and eyes directed outwards. | Enlarged glands | [5] |
| *X. frontalis* | Non-resource defence by hovering and turning around own body axis. |  | [6] |
| X. g*ualanensis* | Territories above the vegetation during the afternoon and includes scent marking at specific locations on plants. | Secretions from the mesosomal glands | [4 ] |
| X. hirsutissima | Males hover close to specific twigs on bushes on mountain tops. Males then make a 2 m excursion flight, often returning to hovering position. Males react to “stone-throwing” only when pheromone is used. | Enlarged glands | [7] |
| *X. micans* | Early in the season males defend territories around floral resources. Late in the summer, males defend landmark territories. They fly horizontal figure-of-eight patterns 1.8x0.6 to 3.8x1.2m in size. Males fly sideways with head always facing outwards. | Secretions from the mesosomal glands | [8] |
| *X. nigrita* | Males circle bushes not in flower and occasionally land; no visible scent marking was seen. | Normal-sized eyes | [5] |
| *X. (Koptortosoma) ogasawarensis* | Territorial behaviour of males at flowering *Scaevola sericea* plants where they hover close to inflorescences. |  | [9 ] |
| X. sulcatipes | Males either have own territories near a flowering bush or a group of males hover around the same bush. | Normal sized eyes; enlarged mesosomal gland | [6, 10] |
| *X. torrida* | Males circle bushes without landing. | Modified legs that grip females; enlarged eyes | [5] |
| *X. varipuncta* | One or several males fly in figure-of-eight or in elliptical paths over small areas, 1–2 ms in diameter, over the tops of plants on hilltops. Non-nest locations, often without nectar or pollen bearing flowers in the vicinity are chosen by males.  Males hover over landmark territories along ridgelines on summer afternoons, along with the use of scent-marks. | Secretions from the mesosomal glands | [11, 12, 13] |
| *X. violacea* | Patrolling flights by males seen commonly. Males alight on high and sunny perches, sit erect with the antennae wide apart, then after 20–40 seconds start grooming or initiate territorial flights, which are slower than patrolling flights and last for less than one minute. Bees would leave the perch to follow other male intruders and other flying insects. Area of these territories ranged from 4–38 m^2^. Territories were located in isolated fields of *Vicia faba* or in areas linking two foraging areas. | Normal-sized eyes; small mesosomal glands | [14, 15] |
| *X. virginica* | Males defend small territories (0.03 m^3^) at nest entrances. They patrol and defend territories of larger size at food plants or near landmarks such as boulders. | Normal-sized eyes; small mesosomal glands | [14, 16] |

**Literature Cited**

1. Leys R (2000) Mate locating strategies of the green carpenter bees *Xylocopa* (Lestis) *aeratus* and *X*. (L.) *bombylans*. J Zool 252: 453–462.
2. Alcock J (1991) Mate-locating behavior of *Xylocopa* *californica* *arizonensis* Cresson (Hymenoptera: Anthophoridae). J Kansas Entomol Soc 64: 349–356.
3. Janzen DH (1966) Notes on the behavior of the carpenter bee *Xylocopa* *fimbriata* in Mexico (Hymenoptera: Apoidea). J Kansas Entomol Soc 39: 633–641.
4. Vinson SB, Frankie GW (1990) Territorial and mating behavior of *Xylocopa* *fimbriata* F. and *Xylocopa* *gualanensis* Cockerell from Costa Rica. J Insect Behav 3: 13–32.
5. Anzenberger G (1977) Ethological study of African carpenter bees of the genus *Xylocopa* (Hymenoptera, Anthophoridae) Z Tierpsychol 44: 337–374.
6. Gerling D, Velthuis HHW, Hefetz A (1989) Bionomics of the large carpenter bees of the genus *Xylocopa*. Ann Rev Entomol 34: 163–190.
7. Velthuis HHW, Camargo JMF (1975) Observations on male territories in a carpenter bee, *Xylocopa* (Neoxylocopa) *hirsutissima* Maidl (Hymenoptera, Anthophoridae). Z Tierpsychol 38: 409–418.
8. Frankie GW, Vinson SB, Lewis A (1979) Territorial behavior in male *Xylocopa* *micans* (Hymenoptera: Anthophoridae). J Kansas Entomol Soc 52: 313–323.
9. Sugiura S (2008) Male territorial behaviour of the endemic large carpenter bee, *Xylocopa* (Koptortosoma) *ogasawarensis* (Hymenoptera: Apidae), on the oceanic Ogasawara Islands. Eur J Entomol 105: 153–157.
10. Velthuis HHW, Gerling D (1980) Observations on territoriality and mating behaviour of the carpenter bee *Xylocopa* *sulcatipes*. Entomol Exp Appl, 28: 82–91.
11. Marshall LD, Alcock J (1981) The evolution of the mating system of the carpenter bee *Xylocopa* *varipuncta* (Hymenoptera: Anthophoridae). J Zool 193: 315-324.
12. Minckley RL, Buchmann SL (1990) Territory site selection of male *Xylocopa* (Neoxylocopa) *varipuncta* Patton (Hymenoptera: Anthophoridae). J Kansas Entomol Soc 63: 329–339.
13. Alcock J (1996) Timing of mate-locating by males in relation to female activity in the carpenter bee *Xylocopa* *varipuncta* (Hymenoptera: Apidae). J Insect Behav 9: 321–328.
14. Leys R, Hogendoorn K (2008) Correlated evolution of mating behaviour and morphology in large carpenter bees (*Xylocopa*). Apidologie 39: 119–132.
15. Vicidomini S (1997) Biology of *Xylocopa* (Xylocopa) *violacea* (Linnaeus, 1758) (Insecta, Hymenoptera, Apidae): male sexual behaviours. II. Atti Mus civ Stor nat Morbegno 8: 95–113.
16. Barrows EM (1983) Male territoriality in the carpenter bee *Xylocopa virginica* *virginica*. Anim Behav 31: 806–813.
